# Endometrial Exosomes/Microvesicles in the Uterine Microenvironment: A New Paradigm for Embryo-Endometrial Cross Talk at Implantation

**DOI:** 10.1371/journal.pone.0058502

**Published:** 2013-03-13

**Authors:** York Hunt Ng, Sophie Rome, Audrey Jalabert, Alexis Forterre, Harmeet Singh, Cassandra L. Hincks, Lois A. Salamonsen

**Affiliations:** 1 Prince Henry's Institute of Medical Research, Melbourne, Victoria, Australia; 2 Laboratory CarMeN (Inserm 1060, INRA 1235, INSA), University of Lyon, Faculté de Médecine Lyon-Sud, Oullins, France; University of Hawaii at Manoa, John A. Burns School of Medicine, United States of America

## Abstract

Exosomes are nanoparticles (∼100 nm diameter) released from cells, which can transfer small RNAs and mRNA via the extracellular environment to cells at distant sites. We hypothesised that exosomes or the slightly larger microvesicles (100–300 nm) are released from the endometrial epithelium into the uterine cavity, and that these contain specific micro (mi)RNA that could be transferred to either the trophectodermal cells of the blastocyst or to endometrial epithelial cells, to promote implantation. The aim of this study was to specifically identify and characterise exosomes/microvesicles (mv) released from endometrial epithelial cells and to determine whether exosomes/mv are present in uterine fluid. Immunostaining demonstrated that the tetraspanins, CD9 and CD63 used as cell surface markers of exosomes are present on the apical surfaces of endometrial epithelial cells in tissue sections taken across the menstrual cycle: CD63 showed cyclical regulation. Exosome/mv pellets were prepared from culture medium of endometrial epithelial cell (ECC1 cells) and from uterine fluid and its associated mucus by sequential ultracentifugation. Exosomes/mv were positively identified in all preparations by FACS and immunofluorescence staining following exosome binding to beads. Size particle analysis confirmed the predominance of particles of 50–150 nm in each of these fluids. MiRNA analysis of the ECC1 cells and their exosomes/mv demonstrated sorting of miRNA into exosomes/mv: 13 of the 227 miRNA were specific to exosomes/mv, while a further 5 were not present in these. The most abundant miRNA in exosomes/mv were hsa-miR-200c, hsa-miR-17 and hsa-miR-106a. Bioinformatic analysis showed that the exosome/mv-specific miRNAs have potential targets in biological pathways highly relevant for embryo implantation. Thus exosomes/mv containing specific miRNA are present in the microenvironment in which embryo implantation occurs and may contribute to the endometrial-embryo cross talk essential for this process.

## Introduction

Establishment of pregnancy requires close developmental synchrony between the endometrium and the blastocyst. Functional interaction between these occurs both during the pre-implantation phase of implantation and during placental development [1]. Implantation is initiated within the microenvironment of uterine fluid which contains a rich array of nutrients, proteins, lipids and other molecules, arising from the endometrium, and probably also from the Fallopian tubes, by selective transudation from blood and, in a conception cycle, from secretions (including human chorionic gonadotrophin (hCG)) from the blastocyst. A number of endometrial secreted factors identified in uterine fluid, can influence endometrial epithelial adhesion molecules, blastocyst outgrowth and other functionalities at implantation [2], while hCG acts at least in part by enhancing epithelial production of a select group of these factors [3], [4], [5].

Exosomes are preformed, membrane-covered vesicles (30–150 nm) of endocytic origin secreted by most cell types *in vitro*, including extravillous and villous trophoblast cells [6] and primary trophoblast from term placenta [7]. They have been identified *in vivo* in all body fluids including amniotic fluid, urine, and blood [8]. Exosomes bear surface receptors/ligands of the original cells and have the potential to selectively interact with specific target cells [9]. In addition to numerous lipids and proteins, exosomes also contain mRNAs and miRNAs [10], [11], [12]. Previous studies have demonstrated that exosomes can horizontally transfer mRNAs to other cells, which can then be translated into functional proteins in the new location [10], [12], [13]. Similarly, miRNAs can be transferred by an exosomal route and further exert gene silencing in the recipient cells [10], [14], [15], [16]. These findings shed new light on the physiological relevance of secretory genomic information by exosomes, and indicate a role of exosomes as new mediators of intercellular cell signaling between neighbouring cells and also between distant tissues, which could act independently but synergistically with soluble growth factors and hormones. Microvesicles, which are slightly larger than exosomes (100–500 nm), are plasma-membrane-derived particles that are also released into the extracellular environment by the outward budding and fission of the plasma membrane. Whether or not these have totally different functions from exosomes or whether there is overlap between the functions of the two particle types is not yet clear, at least in part because all particles contain miRNA and mRNA that are transferable.

It has been proposed that receptivity of the endometrial luminal epithelium could be altered by non-transcriptional, non-translational mechanisms including exchange of signalling and adhesion molecules from endosomal compartments within the cell to and from the cell surface, redistribution of components in the surface between membrane domains, and proteolysis and shedding that might include shedding of exosomes [17]. We therefore hypothesised that the endometrial epithelium releases exosomes into the uterine cavity, where by transfer of their contents to either the blastocyst or adjacent endometrium, they could influence implantation. Disturbance of endometrial exosome release, content or uptake, could contribute to implantation failure as occurs in a number of women presenting with infertility.

MiRNAs are conserved non-coding RNAs of 19–22 nucleotides that function as negative regulators of gene expression, conferring gene silencing by binding target mRNAs in association with the multiprotein RNA induced silencing complex (RISC). Bioinformatic analyses indicate that each miRNA can regulate multiple target RNAs, and that individual mRNA can be targeted by several miRNAs, providing considerable complexity [18]. Recently it has been demonstrated that miRNAs can be selectively transferred from cells into exosomes and thence to other cells [12]. MiRNAs play an essential regulatory role during development, with their levels changing in different cell types and at different developmental stages. Importantly, in the present context, aberration of blastocyst miRNA expression is associated with human infertility [19]. It could be that miRNAs, transferred by an exosomal route within the uterine cavity, contribute to the miRNA content of the blastocyst and/or the endometrium, contributing to their synchronised peri-implantation development and enhancing implantation potential.

We examined human endometrium across the menstrual cycle for exosome membrane marker proteins, then harvested and positively identified exosomes/mv released from an endometrial epithelial cell line, using these markers and size analysis. We then compared endometrial cell miRNA with that present in their exosomes/mv and bioinformatically determined the pathways with potential for regulation by these. Importantly we positively identified exosomes/mv within uterine fluid and its associated mucus, harvested by lavage, supporting that exosomes/mv are indeed present within the intrauterine environment where they could act to modify implantation potential.

## Materials and Methods

### Endometrial tissue, uterine fluid and mucus collection

Ethical approval was obtained for all human sample collections from Human Ethics Committees at Southern Health (# 03066B) and Monash Surgical Private Hospital (# 04056) and written informed consent was obtained from all women. Uterine fluid was obtained by lavage with 2.5 mL of sterile saline, and endometrial tissue obtained by biopsy or at curettage, as previously described [20]. Tissue was classified as normal if the woman was of proven fertility, and without uterine abnormalities such as endometrial polyps, endometriosis or endometritis, or who had received steroid hormone treatment in the last six months. Menstrual cycle phase was confirmed by histological dating [21]. Tissue was fixed in 10% buffered formalin, washed three times in Tris-buffered saline (TBS, pH 7.6) and processed to wax. Uterine fluid was centrifuged gently to remove cellular debris. The supernatant was held at 4 C while mucus within the sample was retrieved and suspended in 1 ml of phosphate-buffered saline (PBS, pH 7.6) (Life Technologies, Grand Island, NY, USA). To release exosomes, the mucus was physically dissociated by vigorous vortexing for 5 min. The supernatant was harvested, and the procedure repeated several times, each in 2–3 ml of PBS (supernatants combined) until the mucus was fully dissociated. Each uterine fluid sample and supernatant from mucus dissociation was then subjected to sequential centrifugation to harvest microvesicles (including exosomes) as described below.

### Endometrial epithelial cell culture

ECC-1, a cell line which closely represents human endometrial luminal epithelium (American Type Culture Collection (ATCC), Rockville, MD, USA) was used for these studies. Cells (6–10×175 cm^2^ flasks (Thermo Scientific, Roskilde, Denmark) were initially cultured in 15 ml DMEM ∶ F12 (v/v 1∶1) medium (Invitrogen, Carlsbad, CA, USA) with added L-glutamine (Life Technologies, Grand Island), 100 U/ml penicillin, 100 µg/ml streptomycin (Commonwealth Serum Laboratory, Melbourne, Australia) and 10% fetal calf serum (Life Technologies). At 70% confluence, they were serum starved for 24 h in 13 ml serum-free DMEM ∶ F12 medium as above but with added transferrin (10 µg/ml), selenium (25 µg/L), bovine serum albumin (BSA) (1 mg/ml) (all from Sigma Diagnostics, St. Louis, MO, USA), linoleic acid (4.7 µg/ml; BD Biosciences, San Jose, CA, USA) and insulin (5 µg/ml; Actrapid, Novo-Nordisk, Sydney, Australia). (TSLI+alb). Medium was harvested, centrifuged at 300 g to remove gross cellular debris and stored at −80 C until further processing.

### Exosome/mv purification and isolation

Exosomes/mv were isolated and purified using a published protocol [22]. Culture medium from ECC-1 cells, uterine fluid or mucus supernatant in PBS, were subjected to differential centrifugation at 4°C (300× g, 10 min to remove cells; 2000× g, 10 min to remove dead cells; 10, 000× g, 30 min to remove cell debris, macroparticles and apoptotic bodies) in 29×104 mm centrifuge tubes (Beckman, Palo Alto, CA, USA) using a Beckman Coulter High Speed Centrifuge Avanti™ J-30I. The supernatants were then ultracentrifuged at 100, 000× g for 70 min in 14×95 mm ultra-clear centrifuge tubes (Beckman) using a Beckman Coulter Ultra High Speed Centrifuge Optima™ L-90K. The pellets from a single sample were pooled, resuspended in PBS and again centrifuged at 100, 000× g for 70 min. Each pellet was finally resuspended in 30 µl of PBS. Any exosomes would be contained in these pellets, along with microvesicles and possibly some apoptotic bodies.

### Quantifying exosome pellets

Purified exosomes/mv (2 µl) were quantified using Nanodrop spectrophotometer, ND-1000 (Thermo-Fisher, Waltham, MA, USA) at an absorbance of 280 nm. PBS was used as the blank.

### Preparation of exosomes/mv for fluorescence-activated cell sorting (FACS) analysis and immunofluorescence (IF)

Since exosomes/mv are too small for direct FACS analysis, they were bound to latex beads by a published method [22]. In brief, 30 µg of purified exosomes/mv were incubated with 10 µl of 4 µm aldehyde/sulfate latex beads (Life Technologies) in 30 µl final volume of PBS at room temperature (RT) for 15 min. 170 µl of PBS was then added, and the mixture incubated on a test tube rotator for 2.5 hours at RT. Then 110 µl of 1M glycine was added (to block the unbound area of the latex beads), and incubation continued for 30 min at RT. The beads were pelleted by centrifugation at 1000 g for 3 min at RT, washed twice with 1 ml PBS/0.5% BSA and the exosome-bead complex incubated with anti-CD9 (MEM-61, Thermo Fisher Scientific, Rockford, IL, USA) or anti-CD63 (MEM-259, Thermo Fisher Scientific) fluorescein isothiocyanate (FITC)-conjugated primary antibodies at RT for 1 h or with anti-CD81 (M-38, Abcam, Cambridge, UK) phycoerythrin (PE) conjugate at 4C overnight. The labelled exosome-bead complexes were again pelleted and washed twice as above. The final complexes were resuspended in 150 µl PBS/0.5% BSA (for FACS analysis) or 10 µl PBS/0.5% BSA (for IF). FACS analyses were performed with a Beckman Coulter MoFlo™ XDP at the Flow Cytometry Facility at Monash Medical Centre, Melbourne, Australia. For immunofluorescence, exosome-latex bead-antibody complexes were each spread on to a microscope slide with a drop of Dakocytomation fluorescent mounting medium (Dako, Carpinteria, CA, USA), air-dried, cover-slipped and sealed with nail polish. The slides were examined using an Olympus BX50 microscope and images taken with an Olympus DP70 camera and DP controller imaging Leica software.

### Immunohistochemistry

Immunohistochemistry for exosome surface markers (CD9 and CD63) was performed on 7 µm sections of normal human endometrium from the proliferative, early secretory and late secretory phases of the menstrual cycle (n = 6/phase). Antigen retrieval used 10 mM citrate buffer (microwave: 3 min high, 10 min med-high) or 50% trypsin/EDTA (15 min at 37C) for CD9 and CD63 respectively, followed by 3% hydrogen peroxide (H_2_0_2_) for 10 min at RT and non-immune block (10% horse serum, 2% human serum at RT for 30 min. Primary antibodies directed against CD 9 (72F6, Abcam) at 1∶600 dilution in 10% horse serum, 2% human serum or CD63 (NK1/C3, Abcam) at 1∶200 dilution, were applied and incubated at 4C overnight. Mouse IgG1 antibody (at individually matched concentration) was used as a negative control for every tissue. Biotinylated horse anti-mouse secondary antibody was applied at RT for 30 min and the sections then incubated with Avidin-Biotin Complex (ABC) (Vector, Burlingame, CA, USA) at RT for 30 min and colour developed with 3,3′-diaminobenzidine (DAB) (Dako) for 3 min. Sections were counter stained with hematoxylin for 30 seconds, followed by acid ethanol (1 second) and lithium carbonate (5 seconds), dehydrated through graded ethanol, cleared with histosol and mounted with DPX mounting medium (Sigma-Aldrich). Each immunostaining run contained quality control sections to ensure consistency of staining between runs. Scoring of the intensity of staining in epithelial cells in each tissue section was performed by two observers, using scores from 1 (pale staining) to 4 (most intense staining).

### Exosome/mv size analysis

Izon's qNano technology (www.izon.com) was employed to detect the size of particles in 100, 000 g pellets from ECC-1 cell culture medium, uterine fluid and mucus. The detector records the particle blockade rate while the pressure applied across a pore sensor is varied [23]. In practice it enables accurate particle-by-particle characterization of vesicles from 50 nm to greater than 1 µm in size in complex mixtures, without averaging the particle sizes.

### Total RNA isolation

Total RNA was isolated from ECC-1 cells (n = 3 separate cultures) with the RNeasy minikit (Qiagen, Inc., Valencia, CA) according to the manufacturer's protocol. The isolated total RNA was treated with DNase using a DNA-free kit (Ambion, Austin, TX, USA) to remove possible genomic DNA contamination. Total RNA from ECC-1 cell derived exosomes/mv (n =  3 separate preparations) were purified by using TriPure Isolation Reagent (Roche Applied Science, Castle Hill, NSW, Australia). The concentrations of total RNA were measured by densitometry (260/280 nm) with a Nanodrop spectrophotometer, ND-1000 (Thermo-Fisher).

### Quantification of mature miRNAs

Mature miRNAs were measured in triplicate using TacMan® Low Density Arrays V3 with the Applied Biosystems 7900HT Fast Real-time PCR system [24]. The Megaplex set pool primers RT human V3 was used to reverse transcribe 30 ng of total RNA extracted either from exosomes or cells. Then a step of pre-amplification was performed by using TaqMan® PreAmp Master Mix designed to preamplify small amounts of cDNA without introducing amplification bias to the sample. Diluted preamplified products (1∶5) were then used as template for PCR reaction by using TaqMan Gene Expression Assay and loaded into the corresponding fill port. Individual single plex PCR reactions were carried out in 384-well plates. The level of miRNA expression was measured using Ct (threshold cycle) determined by RQ Manager. Each array included 3 TaqMan miRNA endogenous controls and one Taqman miRNA assay not related to human.

For each miRNA, the *C*t was calculated by the ABI 7900 Sequence Detection System software. Raw *C*t values considered “undetermined” by the software or at a level ≥40 cycles, were excluded from analysis. For each TaqMan Low Density Array, quality controls were performed on the raw data by checking internal controls and using box plot diagrams. Since the currently used normalisation factor mammU6 plotted in each card was not stably expressed in our different samples we used the mean expression level of expressed miRNAs for normalization [25].

### Statistical and bioinformatic analyses

The semi-quantitative data from immunohistochemistry was tested for normality, followed by one-way ANOVA using GraphPad Prism 5 computer software (GraphPad Inc., San Diego, CA, USA). Significant differences between phases of menstrual cycle were determined using Tukey's test and considered significant when *P*<0.05.

Target genes of the 227 exosomal/mv miRNAs were predicted by bioinformatics using TargetScan 6.1 (http://www.targetscan.org/; includes only miRNA binding sites conserved among species, minimizing the number of false positive target genes). Functional analyses of exosome-secreted miRNA target genes were realized using Babelomics 4.3 (http://babelomics.bioinfo.cipf.es).

## Results

### Immunostaining for exosomal markers on endometrium *in vivo*

**Figure 1:**
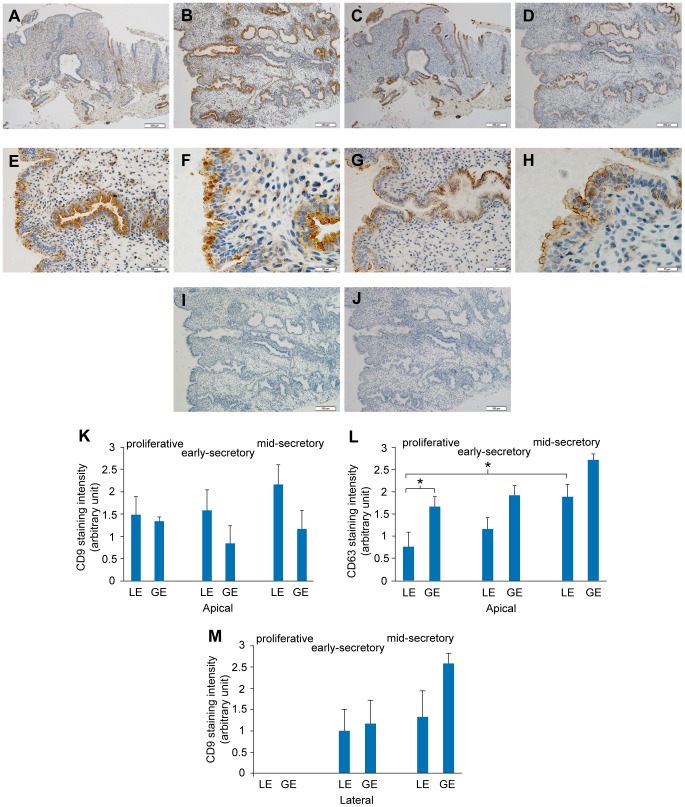
Immunostaining for CD9 and CD63 in endometrium across the menstrual cycle. A–J. Immunostaining for the tetraspanins (CD63. CD9) that were subsequently used as exosomal markers, in endometrial tissues. Endometrial staining for CD63 (plates A,B,E,F) and CD9 (plates C,D,G,H) across the menstrual cycle. Plates A,C: proliferative phase and plates B,D,E–H: secretory phase respectively. Negative controls (Plates I,J). Size bars represent 200 µm (A–D, I–J), 50 µm (E,G), 20 µm (F, H). K–M. Semiquantitative analysis for CD63 (L) and CD9 (K) in luminal epithelium (LE) and glands (GE) in proliferative, early- and mid-secretory phases. Apical (K) and lateral (M) staining for CD9 are presented separately, while CD63 was detected only as apical stain.

Immunolocalisation studies were performed to determine whether the tetraspanins generally used as markers for exosomes, are expressed on the apical surface of endometrial epithelium *in vivo*. If so, these would provide appropriate markers for endometrial exosomes. CD9 was present in all endometrial sections and was clearly detectable on the apical surface of luminal and glandular epithelium (Fig. 1, C, D, E, F) but without cyclical change (Fig. 1 K). Epithelial cytoplasmic positivity was evident in some sections. CD9 was also strongly localized to the lateral membranes, particularly within the glands but only in the secretory phase (Fig. 1, G, H). No lateral staining was observed in proliferative phase samples (Fig. 1C). Leukocytes also stained strongly for CD9. The combined CD9 data are shown graphically in Fig. 1 (K (apical) and M (lateral)).

CD63 was similarly detected in both glandular and luminal epithelial cells (and some leukocytes) in all endometrial tissues from the proliferative, early- and mid-secretory phases of the cycle (Fig. 1A, B, E, F). Glandular staining was often punctate within the cells (Fig. 1E, F). Apical staining was strong but overall more intense in glands than luminal epithelium (P<0.05 in the proliferative phase) and overall increased from the proliferative through the early to mid-secretory phase (P<0.05, luminal epithelium: proliferative versus secretory) (Fig. 1L). Where present, secretions were often immunoreactive. Some glands were entirely devoid of stain, this being more common in proliferative phase samples (Fig. 1A).

### FACS analysis and immunostaining of 100,000 g particles released by endometrial epithelial cells

**Figure 2:**
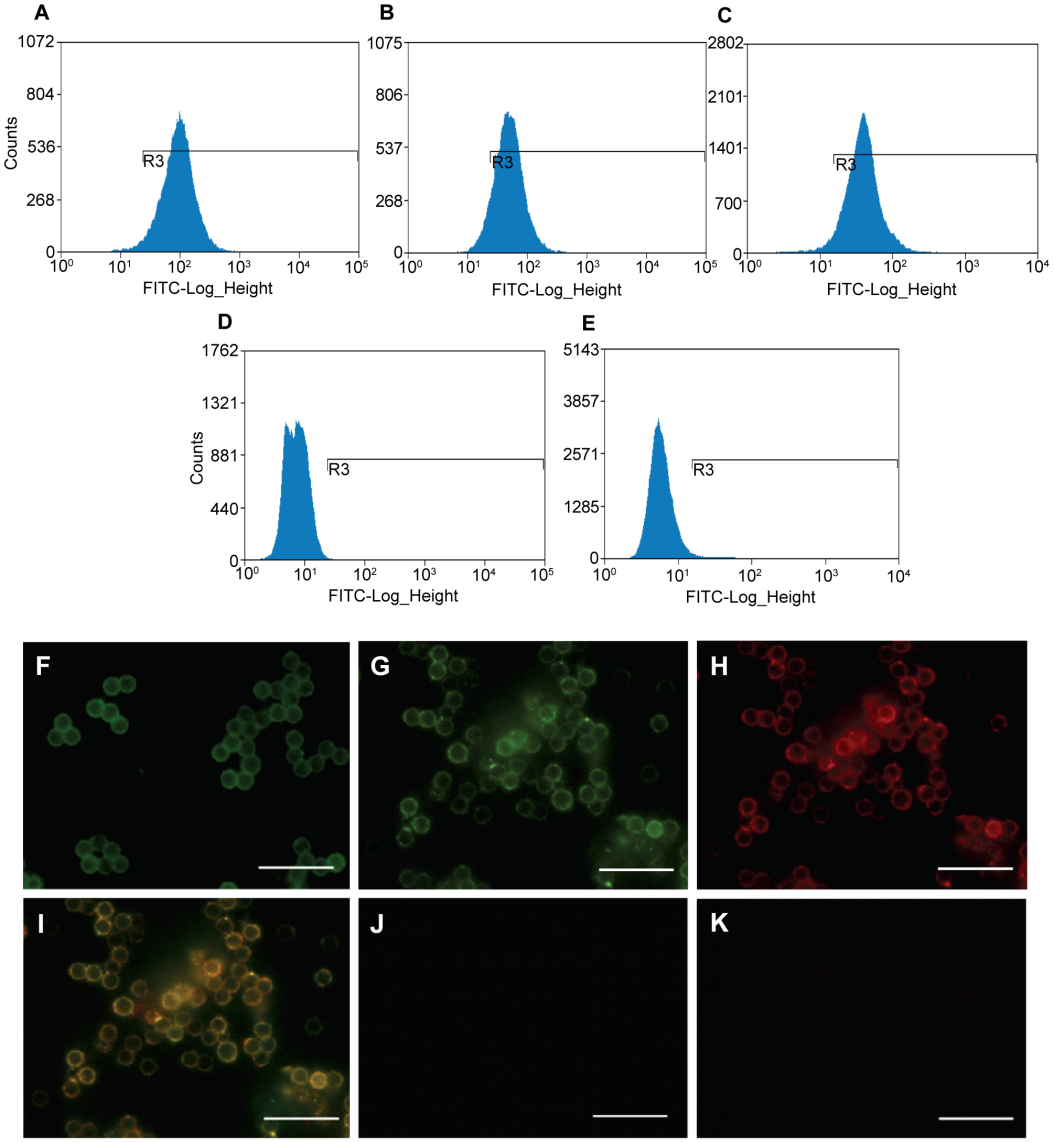
Endometrial exosomes/microvesicles are identified by FACS analysis when bound to latex beads. A–E. Appropriate gating demonstrated strong intensity for each of the exosomal surface proteins, CD9, CD63 and CD81 (Figs. 2A, B, C respectively). Controls - beads alone similarly incubated with isotope specific FITC or PE-labelled IgG (Figs. 2D, E respectively). F–K. Immunostaining of the same beads clearly visualised these same markers on the surface of the beads (F–H respectively): this staining colocalized for CD63 and CD81 shown in I. Negative controls (J,K) (bars, 20 µm).

Given that exosomes/mv are too small for direct FACS analysis, they were first bound to latex beads, then visualized following separate incubation with fluorescence-conjugated antibodies to the exosome markers CD9 and CD63 (FITC- label, Fig. 2A, B respectively) and CD81 (PE-label) (Fig. 2C). Controls were exosome-beads similarly incubated with isotype-specific FITC or PE-labelled IgG. (Fig. 2D, E respectively). Appropriate gating demonstrated strong intensity for each of the surface proteins. Immunostaining of the same beads clearly visualised these same markers on the surface of the beads (Fig. 2, F–H representing CD9, CD63, CD81 respectively; merged stain for CD63 and CD81 shown in Fig. 2I). Negative controls for FITC and PE are shown in Fig. 2J, K respectively). These combined data verify that exosomes/mv released by ECC1 cells, bear these markers. Additional negative controls (antibody plus beads alone) showed no staining in all cases (data not shown). Experiments were repeated with three independent exosome/mv preparations with similar results.

### Exosome identification in uterine fluid and associated mucus

**Figure 3:**
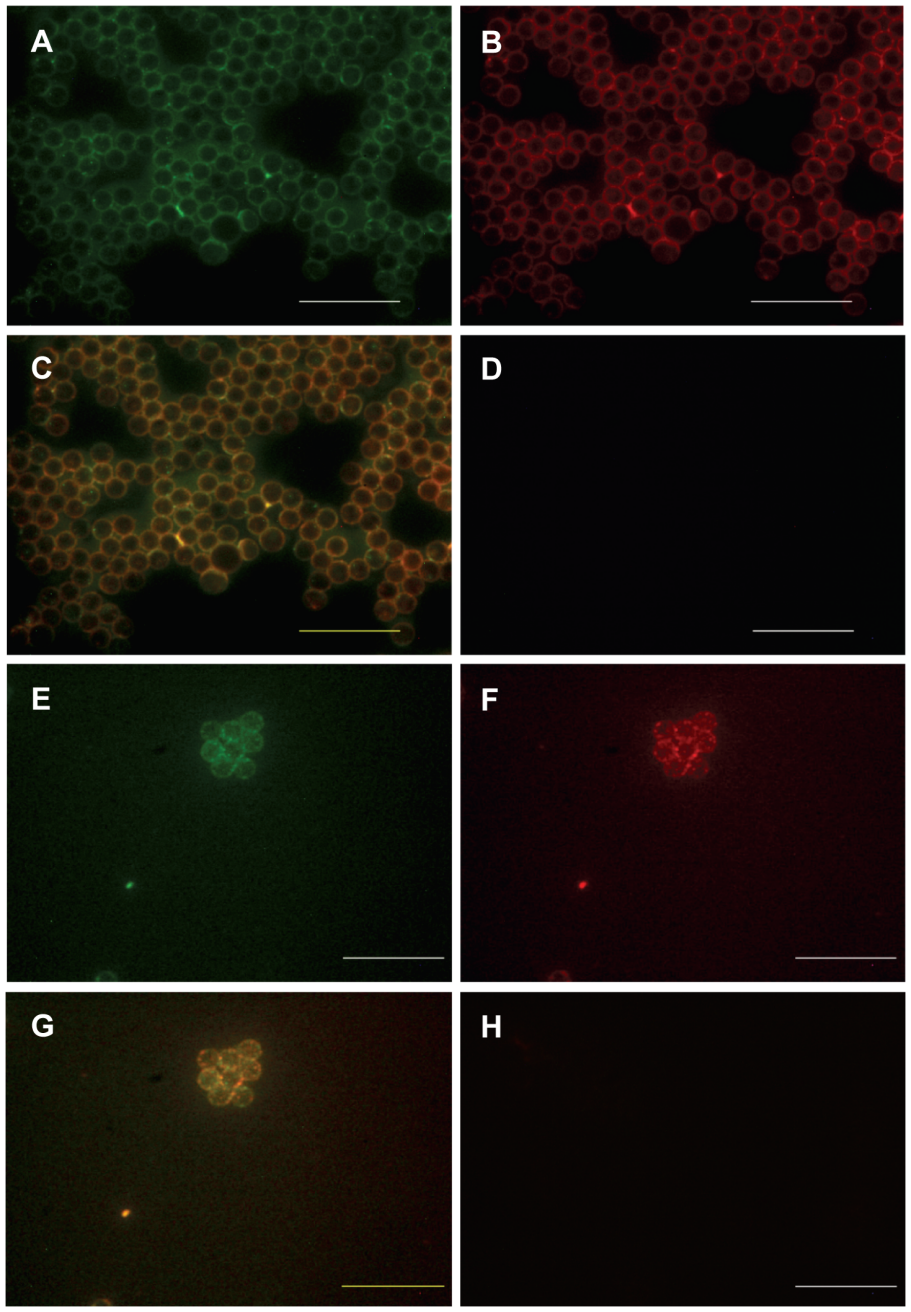
Positive staining for exosome markers on nanoparticles isolated from uterine fluid and associated mucus. Exosome markers CD63 (A,E) and CD81(B,F) positively identify exosomes in the 100,000 g pellet from (a) uterine fluid (A–D) and (b) in the 100,000 g pellet following dissociation of mucus (E–H). All exosomes are bound to latex beads. Merged staining co-localises these markers (C, G) Negative controls have normal IgG replacing primary antibody (D, H). n = 5 separate samples examined for each of fluid and mucus.

The 100,000 g pellets obtained from uterine lavage samples and from associated mucus, were similarly incubated with latex beads followed by specific antibodies to CD63 and CD81. These were visualized by immunofluorescence. Positive staining for both CD63 (Fig. 3A, E), and CD81 (Figs. 3B, F), demonstrated exosomes/mv derived from both uterine fluid (n = 5) (Fig. 3A, B,) and associated mucus (n = 5) (Fig. 3E, F). Further confirmation of the presence of exosomes/mv was by merged staining (Fig. 3C, G for uterine fluid and mucus respectively). Negative controls are shown in Fig. 3D, H.

### Size analysis of exosomal preparations

**Figure 4:**
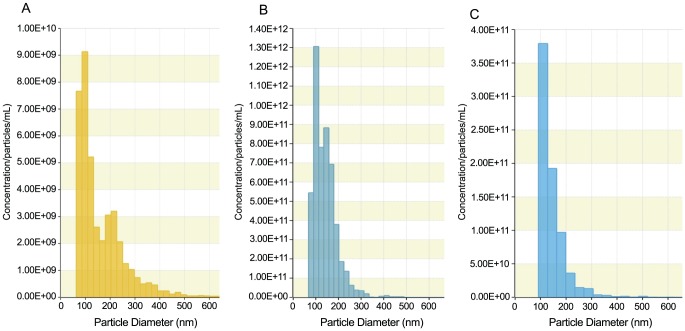
Size analysis confirms exosomal/microparticle identity of positively stained particles. Representative particle diameters of 100,000 g pellets from (A), ECC1 culture medium, (B) uterine fluid, (C) dissociated mucus. [Exosomes are 30–150 nm, microvesicles are 100 nm–1 µm and apoptotic bodies are 500 nm–3 µm in diameter].

Three main types of vesicles are released by cells: apoptotic bodies (500 nm–3 µm in diameter) released by cells undergoing apoptosis; shedding microvesicles that bud from the plasma membrane (100 nm–1 µm) and exosomes that are released by exocytosis from multivesicular bodies of the endosome (stated variously as 30–100 or 30–150 nm) [6], [26]. The size distribution of such particles in our preparations was measured by nanoparticle tracking analysis. Representative distributions for ECC1 cell-derived vesicles and for those obtained from uterine fluid and associated mucus are shown in Fig. 4A, 4B and 4C respectively. In each case 3 separate preparations were analysed with very similar results. Very few particles of >500 nm were detected in any preparation, thus excluding apoptotic bodies as major components of the samples. In A and B, the major peak of particle distribution fell between 30–100 nm. The next greatest distribution (the major distribution in C) was between 100–150 nm. Thus all samples (ECC1 cells, uterine fluid, mucus; Fig. 4A–C respectively) contain a highly enriched mixture of exosomes and microvesicles.

### Profiling of miRNAs in endometrial epithelial cells exosomes/mv

**Figure 5:**
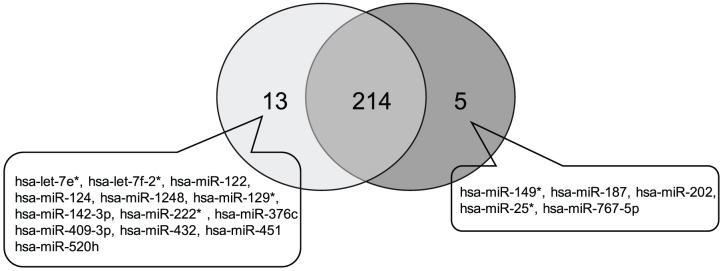
Venn diagram comparing the miRNA profile of exosomes versus ECC1 parent cells and showing the number of shared and specific miRNAs.

To avoid selection of false-positive miRNAs, results were included only if the miRNA was commonly expressed in all three biological replicates each of ECC1 cells and exosomes/mv. The number of miRNAs profiled was similar in the ECC1 cells and exosomes/mv (219 and 227 respectively) (Fig. 5), representing approximately 31% of the miRNAs on the arrays. Of these, 214 were common to both exosomes/mv and cells, while 13 miRNAs were specific to the exosomes/mv and 5 unique to the cells (Fig. 5 and Table 1). These data demonstrate sorting of certain miRNAs into the microparticles. In addition, ECC-1 derived exosomes/mv also contained the non-coding small nuclear RNA U6 involved in the spliceosome, and RNU44 and RNU48 which are small RNA molecules that primarily guide chemical modifications of other RNAs. The complete list of miRNAs identified is provided in Table S1.

**Table 1:** miRNAs selectively expressed in ECC1 cells or ECC1 exosomes.

EXOSOMES	ECC-1 Cells
hsa-let-7e*-002407	hsa-miR-149*-002164
hsa-let-7f-2*-002418	hsa-miR-187-4373307
hsa-miR-122-4395356	hsa-miR-202-4395474
hsa-miR-124-4373295	hsa-miR-25*-002442
hsa-miR-1248-002870	hsa-miR-767-5p-001993
hsa-miR-129*-002298	
hsa-miR-142-3p-4373136	
hsa-miR-222*-002097	
hsa-miR-376c-4395233	
hsa-miR-409-3p-002332	
hsa-miR-432-001026	
hsa-miR-451-4373360	
hsa-miR-520h-001170	

The 20 most highly expressed miRNAs in each case are listed in Table 2. Of these, 16 were common to both cells and exosomes/mv: most of the others were of lower relative abundance in one or the other. The *C*t values for the highly abundant miRNA, (hsa-miR-200c) in both cells and exosomes/mv were 21.3±0.14 and 19.0±0,04 (mean ± SD) respectively. The *C*t values were >35 for 2 exosomal/mv miRNAs (has-miR-29c and -628-5p) and for 3 cellular miRNAs (has-miR-29c, -372, and -645).

**Table 2:** Highest 20 expressed miRNA in exoxomes and ECC1 cells.

EXOSOMES	Ct value (3 independent exosome preparations)		
hsa-miR-200c-4395411	21.3155448	21.1605498	21.4502582
hsa-miR-17-4395419	22.3041065	22.1451368	22.4117373
hsa-miR-106a-4395280	22.3102025	22.1562335	22.4088089
hsa-miR-30c-4373060	22.3142664	22.1269784	23.4034762
hsa-miR-222-4395387	22.3162984	22.1572423	22.4029522
hsa-miR-484-4381032	22.3183304	23.1690669	22.4029522
hsa-miR-19b-4373098	22.3234104	22.1632951	22.4136896
hsa-miR-526b-4395493	22.3254424	23.1287149	22.3863582
hsa-miR-24-4373072	22.3356023	22.1663215	22.4419971
hsa-miR-191-4395410	22.3366183	22.1653127	21.4453776
hsa-miR-92a-4395169	23.3485479	24.208129	23.393715
hsa-miR-30b-4373290	23.3495639	23.1640229	23.4112852
hsa-miR-197-4373102	23.3505799	25.2320591	23.4034762
hsa-miR-200b-4395362	23.3505799	23.1609965	23.4073807
hsa-miR-342-3p-4395371	23.3546438	24.2000586	22.3961194
hsa-miR-193b-4395478	23.3566758	23.1872252	22.4263791
hsa-miR-99b-4373007	23.3668358	24.2111553	23.3605269
hsa-let-7e-4395517	23.3881716	23.1751197	22.4302836
hsa-let-7b-4395446	23.3922356	24.1748386	22.4419971
hsa-miR-574-3p-4395460	24.3462535	26.2065581	24.3776449

ECC1-cells	Ct value (3 independent ECC1-cell preparations		
hsa-miR-200c-4395411	18.9407079	19.0060113	18.9363572
hsa-miR-17-4395419	19.9435866	20.9942093	20.9563685
hsa-miR-106a-4395280	19.956585	20.9942093	19.9393698
hsa-miR-191-4395410	19.9695834	20.0051158	19.9673422
hsa-miR-222-4395387	20.9424657	20.973186	21.9463939
hsa-miR-197-4373102	20.9694624	20.9771905	20.9603646
hsa-miR-484-4381032	20.9714622	20.991206	20.9443804
hsa-miR-193b-4395478	20.9844606	20.9982137	20.9543705
hsa-let-7e-4395517	21.006458	21.0192369	20.9623626
hsa-let-7b-4395446	21.0124572	21.0232414	20.9024217
hsa-miR-24-4373072	21.0274554	20.9952104	20.9613636
hsa-miR-1233-002768	21.9533695	21.0844306	21.9129706
hsa-miR-99b-4373007	21.9583428	21.9752939	21.9663742
hsa-miR-200b-4395362	21.9793402	22.0153381	21.9893516
hsa-miR-342-3p-4395371	21.98134	21.9983193	21.9364037
hsa-miR-92a-4395169	21.9833397	22.0073293	21.952388
hsa-miR-149*-002164	22.0023815	23.0220056	22.8739784
hsa-miR-16-4373121	22.9472231	22.9914172	22.9414143
hsa-miR-149-4395366	22.9532224	22.9904161	22.9524035
hsa-let-7a-4373169	22.9652209	23.0434748	22.9683877

### Potential target genes for exosomal/mv miRNAs

Target genes of the 227 exosome-secreted miRNAs were predicted using TargetScan 6.1 (http://www.targetscan.org/). Among the 227 miRNAs, 189 had predicted target genes (n = 8934 unique genes). Functional analysis of exosome-secreted miRNA target genes was performed using Babelomics 4.3 (http://babelomics.bioinfo.cipf.es). Table 3 shows KEGG pathways that are most significant in terms of containing more genes than expected (*p*<0.05 and adjusted *p*<0.05). A number of these pathways contain genes known to be important for and requiring regulation at implantation. These include adherens junctions, ECM-receptor interactions, the VEGF-signalling pathway, the Jak-STAT pathway and the Toll-like receptor signalling pathway. Metabolic pathways are also highly likely to be of importance. The genes in the adherens junction regulation and ECM-receptor interaction pathways that appear in the potential targets of endometrial exosomes/mv are shown in red in Figs. S1 and S2.

**Table 3:** KEGG pathways significantly enriched in exosomal-miRNAs target genes.

KEGG ID	KEGG pathways	No of target genes	Adjusted p value
hsa04910	Insulin signaling pathway	115	3.26E-20
hsa04120	Ubiquitin mediated proteolysis	105	8.65E-20
hsa04350	TGF-beta signaling pathway	86	8.65E-20
hsa04520	Adherens junction	81	8.65E-20
hsa04012	ErbB signaling pathway	81	1.10E-17
hsa04530	Tight junction	109	1.09E-16
hsa04660	T cell receptor signaling pathway	89	3.17E-16
V	Adipocytokine signaling pathway	66	3.17E-16
hsa04512	ECM-receptor interaction	75	1.83E-15
hsa04114	Oocyte meiosis	91	8.91E-15
hsa04912	GnRH signaling pathway	85	9.06E-15
hsa04110	Cell cycle	85	3.98E-14
hsa04070	Phosphatidylinositol signaling system	69	4.18E-14
hsa04666	Fc gamma R-mediated phagocytosis	79	1.74E-13
hsa04670	Leukocyte transendothelial migration	90	1.83E-13
hsa04340	Hedgehog signaling pathway	58	2.63E-13
hsa04540	Gap junction	78	1.22E-12
hsa04142	Lysosome	90	2.17E-12
hsa04914	Progesterone-mediated oocyte maturation	70	3.76E-12
hsa00562	Inositol phosphate metabolism	51	1.43E-11
hsa04370	VEGF signaling pathway	64	1.81E-11
hsa05213	Endometrial cancer	54	2.07E-11
hsa00564	Glycerophospholipid metabolism	57	2.56E-11
hsa04150	mTOR signaling pathway	53	3.44E-11
hsa04664	Fc epsilon RI signaling pathway	64	7.80E-11
hsa00510	N-Glycan biosynthesis	46	2.28E-10
hsa00230	Purine metabolism	90	3.87E-10
hsa04210	Apoptosis	68	5.71E-10
hsa04115	p53 signaling pathway	60	2.30E-09
hsa04330	Notch signaling pathway	51	3.18E-09
hsa04662	B cell receptor signaling pathway	59	3.63E-09
hsa00520	Amino sugar and nucleotide sugar metabolism	40	6.75E-09
hsa04960	Aldosterone-regulated sodium reabsorption	38	1.32E-07
hsa04130	SNARE interactions in vesicular transport	42	3.38E-07
hsa00250	Alanine, aspartate and glutamate metabolism	36	3.79E-07
hsa04630	Jak-STAT signaling pathway	94	4.28E-07
hsa04620	Toll-like receptor signaling pathway	71	9.29E-07
hsa00310	Lysine degradation	42	1.16E-06
hsa03040	Spliceosome	79	3.17E-06
hsa03018	RNA degradation	47	8.11E-06
hsa04622	RIG-I-like receptor signaling pathway	53	9.18E-06
hsa00600	Sphingolipid metabolism	38	2.30E-05
hsa02010	ABC transporters	39	3.82E-05
hsa03320	PPAR signaling pathway	47	4.32E-05
hsa04621	NOD-like receptor signaling pathway	45	1.92E-04
hsa00240	Pyrimidine metabolism	54	3.59E-04
hsa00010	Glycolysis/Gluconeogenesis	37	1.68E-03
hsa00500	Starch and sucrose metabolism	38	3.77E-03
hsa04640	Hematopoietic cell lineage	49	

## Discussion

Transfer of genetic and protein material between cells at a distance via exosomes/mv is a relatively new concept for cell-cell signalling [27], [28]. Here, for the first time, we positively identify that exosomes are present in uterine fluid where they could act to directly transfer such information from the endometrium to a blastocyst in a conception cycle, thus altering the potential for implantation success. The validity of these nanoparticles as exosomes/mv was confirmed by the presence of known surface markers and by size analysis. These markers, CD9 and CD68 were also present on the apical surface of human endometrial epithelium, identifying this as the likely source of the exosomes in the uterine cavity. Furthermore, since primary endometrial epithelial cells are not available in sufficient quantities to provide exosomes for further studies, exosomes/mv derived from the ECC1 cell line, which closely resembles endometrial luminal epithelium [29], [30] were similarly characterised and their miRNA content defined.

Exosomes appear to contribute to a diverse range of biological processes, depending on the cell of origin and the conditions for secretion. Current evidence suggests that exosomes fuse with the plasma membrane of the recipient cell and release their contents into the target cell. Proposed mechanisms are that there is binding at the cell surface via specific receptors [31], or that internalization is by exocytosis [32]. Most studies to date have been performed *in vitro* and with cell lines since very large numbers of cells or large volumes of body fluids are needed to provide sufficient exosomes for experimentation. In the case of uterine fluid which is present only in µl quantities *in vivo*, cell lines such as the ECC1 line used in the present study represent the only feasible source of endometrial exosomes for experimentation.

The trapping of exosomes/mv in mucus contained within the uterine lavage samples could be of physiological relevance. Uterine lavage from which the mucus in our studies was derived, washes the endometrial epithelial surface, which is normally covered by a thick glycocalyx consisting of highly glycosylated mucins including Muc-1 [33] and other cell surface binding molecules including heparin-sulfate proteoglycans such as syndecan [34]. Muc-1 is known to be cleared precisely at the site of blastocyst implantation [35], and it could be that trapped exosomes/mv are released in close proximity to the implantation site, where they could immediately bind to the trophectodermal cells.

While exosomes are now being widely studied in a variety of systems, particularly in relation to cancer, the particle size in their preparations is often not adequately defined. Apoptotic bodies, which are generally of 500–100 nm are a common contaminant and can provide misleading data. The 100,000 g fractions in this study were measured using qNano technology which measures every particle individually and which defined a major peak in each preparation between 50–150 nm, the size of exosomes, but with overlap with microvesicles from 100–150 nm. The tail from these peaks extended to ∼300 nm, suggesting larger microvesicles were also present but in smaller amounts. However, both exosomes and microvesicles contain and deliver genetic material in the form of mRNA and miRNA to recipient cells [28].,

ECC1 cells as used here, have recently been shown to release microvesicles containing the glycosylated transmembrane protein extracellular matrix metalloproteinase (MMP) inducer (EMMPRIN) when stimulated via either estrogen receptor or the G-protein coupled receptor (GPR) 30b [36]. This protein (also known as CD147), may act as a marker of endometrial microvesicles: whether it is functional within the uterine cavity remains to be determined. These microvesicles were harvested at 40,000 g and thus it is not clear that they are the same as the exosomes/mv harvested at 100,000 g in the present study.

The tetraspanins CD9 and CD63, membrane-bound proteins that are commonly used to identify exosomes/microvesicles [37], were used to confirm the identity of the particles in this study. Their expression and cellular localization was first examined across the menstrual cycle and some interesting features noted. Both tetraspanins showed strong apical staining on both luminal and glandular epithelial cells, as anticipated if exosomes were to be shed from these surfaces. Interestingly, the intensity of staining for CD63 increased across the cycle to reach a maximum in the mid-secretory phase, the time of endometrial receptivity for implantation, while at this time CD9 was also localised to lateral membranes. Progesterone is the dominant hormone in the mid-secretory phase, driving the molecular changes required for implantation: it down-regulates CD63 transcription in cultured endometrial stromal cells [38] but there is no data regarding epithelial regulation. Both CD9 and CD63 were also present in leukocytes, likely to be uterine NK cells in which their expression is known [39]. CD9 associates with CD98 in the endometrial epithelium in mice in which form it contributes to implantation success [40], [41]. It has also been also used as a marker of stemness in endometrial epithelium [42]. Whether this component of exosome membranes has any function after exosome uptake by other cells, has not been examined.

CD9 is a member of the transmembrane 4 superfamily, also known as the tetraspanin family. It can modulate cell adhesion and migration and also trigger platelet activation and aggregation. It is thus likely that it is involved in exosome-target recognition [43]. In addition CD9 mediates signal transduction events that play a role in the regulation of cell development, activation, growth and motility. CD9 seems involved in the packaging of proteins in exosomes [44].

Analysis of the miRNA content of the exosomes/mv and of their parent ECC1 cells, demonstrated some sorting of miRNA: this has been reported also for exosomes isolated from murine dendritic cell culture medium [45]. Interestingly in that study, 5 of >200miRNAs were unique if the cells of origin were immature, but 58 unique miRNA were sorted from mature cells. Likewise exosomes derived from myoblast and myotube cells and those from PC-prostate cancer cells [46] contained differentially sorted miRNA [47]. In the present study, the ECC1 cells were not subjected to hormonal stimulation as would occur *in vivo* during the normal menstrual cycle: while the phenol red in culture medium exerts an estrogenic influence, progesterone which induces differentiation of endometrial epithelium, may influence the packaging of miRNA into exosomes. Certainly in entire endometrium of women undergoing IVF in whom high progesterone levels are often found, 4 miRNAs were associated with changes in progesterone; hsa-miR-451, -424, -125b and -30b [48]. Of these, hsa-miR-451 was detected exclusively in exosomes in our study, while hsa-miR-30b was present in both exosomes and ECC1 cells. Future studies examining progesterone effects on endometrial exosomal miRNA are clearly warranted.

Other microRNAs have been shown to be altered in association with endometrial receptivity in intact normal endometrium which contains a number of different cell types (epithelial, stromal, leukocytes, cells of the vasculature). Altmae et al. [49] examined the miRNA signatures of fertile endometrium and compared non-receptive (day LH+2) with receptive (LH+7) biopsies. They found hsa-miR-30b and -30d to be significantly upregulated while hsa-miR-494 and hsa-miR-923 downregulated in receptive endometrium. The two upregulated miRNAs (hsa-miR-30b and -30d) were also detected in our study both in ECC1 cells and in their exosomes: hsa-miR-30b was among the 20 most abundant in exosomes but not in ECC1 cells, suggesting enrichment. Hsa-miR-494 was also present in both exosomes and ECC1 cells while hsa-miR-923 was not detected. The latter appears to be a fragment of 28S rRNA and has now been removed from the majority of databases [49]. Another study on similar biopsies but using deep sequencing [50] identified 20 miRNA significantly changed with receptivity: of these, 8 were upregulated and 12 down-regulated on LH+7. 3 of these were in common with the Altmae study. Importantly bioinformatics showed these miRNAs target a large set of genes that are known to be differentially expressed during the receptive phase. Of their list of receptivity related miRNAs, 9 were not detected in our study of ECC1 cells and are most likely derived from other cell types. However, 9 (hsa-miR-30d, -30b, -31, -193a-5p, -125b, -452, -455-3p, -483-5p, -100) are also present in our study in both ECC1 cells and exosomes. Importantly, 2 (hsa-miR-455-5p, hsa- mir-143) are among our differentially expressed miRNAs appearing in cells but not exosomes.

MiRNAs have also been identified in separate human endometrial epithelial and stromal cells. Interestingly there was a lower number of miRNAs in glandular epithelial cells than stromal cells and these were the same miRNAs as found in the entire tissues from which they were derived [51]. In epithelial cells, estradiol-17β inhibited hsa-miR-21 in epithelial cells while medroxyprogesterone acetate increased hsa-miR20a and hsa-miR-26a. Since these were all present in our cells and exosomes/mv, it will be interesting to see whether they are similarly steroid hormone regulated in our future studies.

Bioinformatics analysis of the exosomal/mv miRNAs in the present study, identified target genes that contribute to many KEGG pathways, a number of which are known as relevant to implantation. For example, ECM-receptor interactions are critical for implantation: these include interactions between fibronectin [52], [53] or osteopontin and integrins [54], [55], [56]. Both adherens and tight junctional proteins including cadherins are tightly regulated at implantation sites [57] since trophectodermal cells must become mobile and penetrate between the endometrial luminal epithelial cells (review [58]). The Jak-STAT pathway is activated by actions of multiple cytokines known for their roles in implantation: IL11 and LIF being two examples [59]. VEGF produced by the epithelium can act on blastocysts, enhancing their outgrowth and adhesive capacity at least *in vitro* demonstrating that the VEGF signalling pathway is present and active in trophoblast [20].

## Conclusions

To our knowledge, this is the first study to identify and examine the presence and biological potential of exosomes/mv in the uterine cavity. Importantly, the identification of exosome/mv specific miRNA has enabled bioinformatic identification of pathways that could be influenced if the exosomes are taken up by trophectoderm or epithelium at the time of implantation, or transferred to sperm as they transit the uterine cavity. Exosomes/mv and/or exosome-derived miRNA or proteins may also prove useful as biomarkers for receptivity or for human endometrial diseases. Given that exosomes have been shown to modulate the behaviour of immune and cancer cells, both of which have actions in common with those of embryo implantation, elucidation of the steroidal regulation and the function of the exosomes in the uterine cavity will extend our understanding of the early embryo-maternal dialogue with potential impacts on our understanding of infertility and success rates of IVF.

## Supporting Information

Figure S1 **KEGG pathway for adherens junctions.** The factors marked in red are potentially regulated by miRNAs present in ECC1 cell-derived exosomes.
(TIF)

Figure S2 **KEGG pathway for ECM-receptor interactions.** The factors marked in red are potentially regulated by miRNAs present in ECC1 cell-derived exosomes.
(TIF)

Table S1 **Complete list of miRNA identified in ECC1 exosomes and cells.**
(DOC)
