# Supplementary figures and images for: Endometrial Exosomes/Microvesicles in the Uterine Microenvironment: A New Paradigm for Embryo-Endometrial Cross Talk at Implantation
Source: PLoS One. 2013 Mar 13;8(3):e58502. doi: 10.1371/journal.pone.0058502 (PMC3596344; doi:10.1371/journal.pone.0058502)

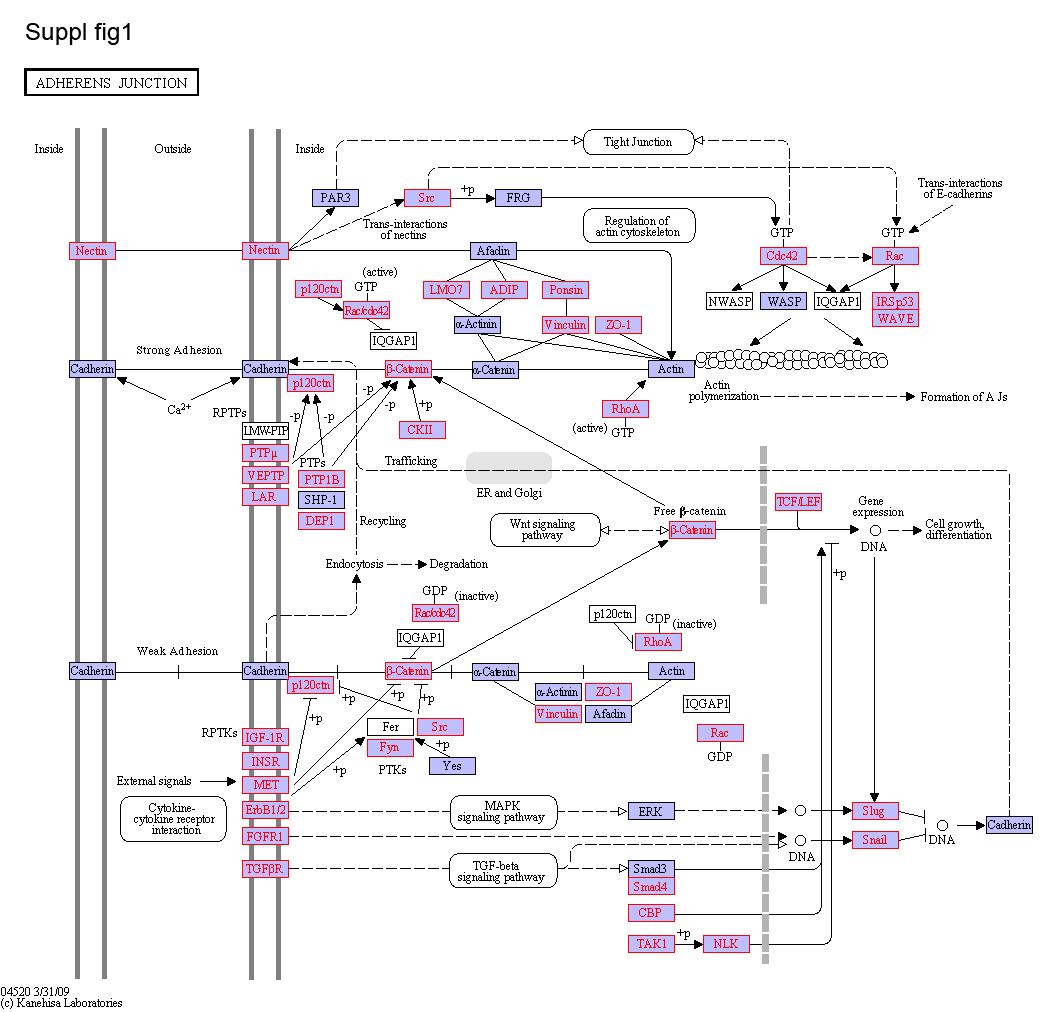

Supplement: Figure S1 — KEGG pathway for adherens junctions. The factors marked in red are potentially regulated by miRNAs present in ECC1 cell-derived exosomes. (TIF) [file pone.0058502.s001.tif]

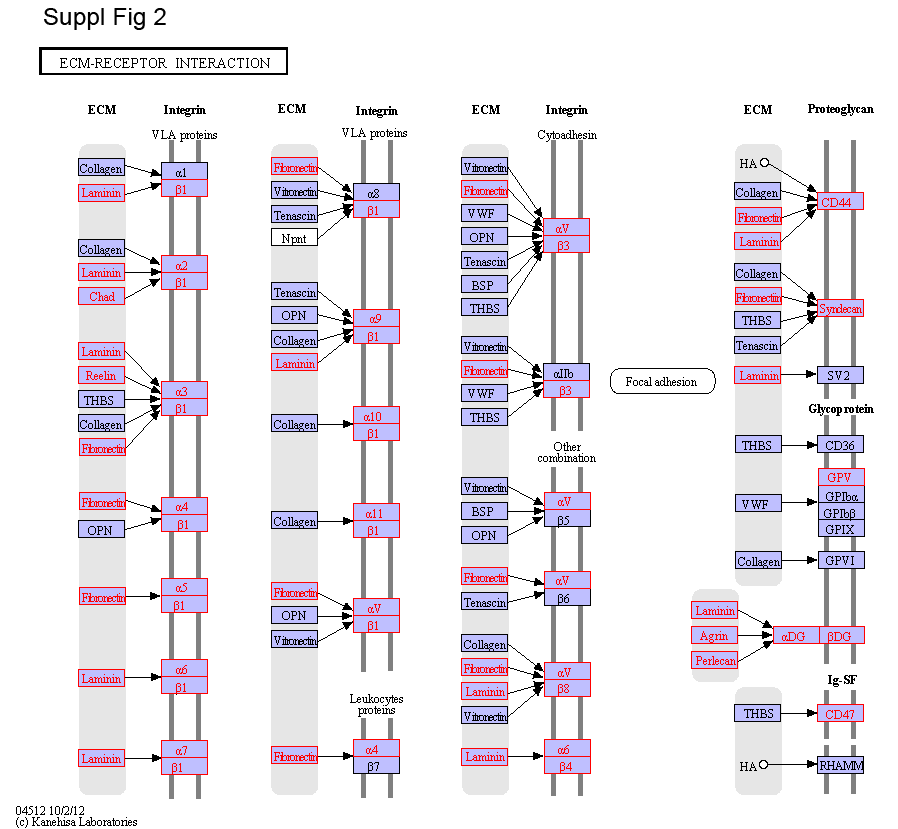

Supplement: Figure S2 — KEGG pathway for ECM-receptor interactions. The factors marked in red are potentially regulated by miRNAs present in ECC1 cell-derived exosomes. (TIF) [file pone.0058502.s002.tif]
