# Supplementary material for: Endometrial Exosomes/Microvesicles in the Uterine Microenvironment: A New Paradigm for Embryo-Endometrial Cross Talk at Implantation
Source: PLoS One. 2013 Mar 13;8(3):e58502. doi: 10.1371/journal.pone.0058502 (PMC3596344; doi:10.1371/journal.pone.0058502)
Supplement: Table S1 — Complete list of miRNA identified in ECC1 exosomes and cells. (DOC) [file pone.0058502.s003.doc]

**Supplementary Table S1.** Complete list of miRNA identified in ECC1 exosomes and cells

| **EXOSOMES** |  | **ECC-1 cells** |
| --- | --- | --- |
| hsa-miR-200c-4395411 |  | hsa-miR-200c |
| hsa-miR-17-4395419 |  | hsa-miR-17 |
| hsa-miR-106a-4395280 |  | hsa-miR-106a |
| hsa-miR-30c-4373060 |  | hsa-miR-191 |
| hsa-miR-222-4395387 |  | hsa-miR-222 |
| hsa-miR-484-4381032 |  | hsa-miR-197 |
| hsa-miR-19b-4373098 |  | hsa-miR-484 |
| hsa-miR-24-4373072 |  | hsa-miR-193b |
| hsa-miR-191-4395410 |  | hsa-let-7e |
| hsa-miR-92a-4395169 |  | hsa-let-7b |
| hsa-miR-30b-4373290 |  | hsa-miR-24 |
| hsa-miR-197-4373102 |  | hsa-miR-99b |
| hsa-miR-200b-4395362 |  | hsa-miR-200b |
| hsa-miR-342-3p-4395371 |  | hsa-miR-342-3p |
| hsa-miR-193b-4395478 |  | hsa-miR-92a |
| hsa-miR-99b-4373007 |  | hsa-miR-149* |
| hsa-let-7e-4395517 |  | hsa-miR-16 |
| hsa-let-7b-4395446 |  | hsa-miR-149 |
| hsa-miR-574-3p-4395460 |  | hsa-let-7a |
| hsa-miR-20a-4373286 |  | hsa-miR-320 |
| hsa-miR-886-5p-4395304 |  | hsa-miR-331-3p |
| hsa-miR-149-4395366 |  | hsa-miR-30c |
| hsa-miR-320-4395388 |  | hsa-miR-125b |
| hsa-miR-331-3p-4373046 |  | hsa-miR-15b |
| hsa-miR-886-3p-4395305 |  | hsa-miR-125a-5p |
| hsa-miR-30a-3p-000416 |  | hsa-miR-19b |
| hsa-let-7c-4373167 |  | hsa-miR-20a |
| hsa-miR-16-4373121 |  | hsa-miR-30b |
| hsa-miR-425-4380926 |  | hsa-miR-30a-3p |
| hsa-miR-28-3p-4395557 |  | hsa-miR-93 |
| hsa-miR-125b-4373148 |  | hsa-miR-126 |
| hsa-miR-100-4373160 |  | hsa-let-7d |
| hsa-miR-15b-4373122 |  | hsa-miR-200a |
| hsa-miR-99a-4373008 |  | hsa-let-7c |
| hsa-miR-218-4373081 |  | hsa-miR-886-5p |
| hsa-miR-151-3p-002254 |  | hsa-miR-20b |
| hsa-miR-30a-5p-000417 |  | hsa-miR-218 |
| hsa-miR-93-4373302 |  | hsa-miR-345 |
| hsa-miR-25-4373071 |  | hsa-miR-30e-3p |
| hsa-miR-126-4395339 |  | hsa-miR-574-3p |
| hsa-miR-223-4395406 |  | hsa-miR-28-3p |
| hsa-miR-345-4395297 |  | hsa-miR-100 |
| hsa-miR-221-4373077 |  | hsa-miR-99a |
| hsa-let-7d-4395394 |  | hsa-miR-210 |
| hsa-miR-1260-002896 |  | hsa-miR-26a |
| hsa-miR-200a-4378069 |  | hsa-let-7g |
| hsa-miR-374b-4381045 |  | hsa-miR-10a |
| hsa-miR-10a-4373153 |  | hsa-miR-1260 |
| hsa-miR-30e-3p-000422 |  | hsa-miR-1275 |
| hsa-miR-10b-4395329 |  | hsa-miR-27b |
| hsa-miR-132-4373143 |  | hsa-miR-766 |
| hsa-let-7a-4373169 |  | hsa-miR-30a-5p |
| hsa-miR-210-4373089 |  | hsa-miR-135b |
| hsa-miR-21-4373090 |  | hsa-miR-425 |
| hsa-miR-9-4373285 |  | hsa-miR-99b* |
| hsa-miR-744-4395435 |  | hsa-miR-25 |
| hsa-miR-31-4395390 |  | hsa-miR-374b |
| hsa-miR-1180-002847 |  | hsa-miR-130b |
| hsa-miR-150-4373127 |  | hsa-miR-744 |
| hsa-let-7g-4395393 |  | hsa-miR-186 |
| hsa-miR-20b-4373263 |  | hsa-miR-106b |
| hsa-miR-19a-4373099 |  | hsa-miR-454 |
| hsa-miR-106b-4373155 |  | hsa-miR-221 |
| hsa-miR-1275-002840 |  | hsa-miR-151-3p |
| hsa-miR-532-5p-4380928 |  | hsa-miR-183* |
| hsa-miR-186-4395396 |  | hsa-miR-532-5p |
| hsa-miR-135b-4395372 |  | hsa-miR-532-3p |
| hsa-miR-183*-002270 |  | hsa-miR-103 |
| hsa-miR-26a-4395166 |  | hsa-miR-181a |
| rno-miR-7*-001338 |  | hsa-miR-93* |
| hsa-miR-625*-002432 |  | hsa-miR-130a |
| hsa-miR-181a-4373117 |  | hsa-miR-27b* |
| hsa-miR-491-5p-4381053 |  | hsa-miR-625* |
| hsa-miR-28-5p-4373067 |  | hsa-miR-324-5p |
| hsa-miR-30d-000420 |  | hsa-miR-196b |
| hsa-miR-145-4395389 |  | hsa-miR-324-3p |
| hsa-miR-141-4373137 |  | hsa-miR-328 |
| hsa-miR-1291-002838 |  | hsa-miR-18a* |
| hsa-miR-320B-002844 |  | hsa-miR-151-5p |
| hsa-miR-130a-4373145 |  | hsa-miR-31 |
| hsa-miR-324-3p-4395272 |  | hsa-miR-1180 |
| hsa-miR-203-4373095 |  | hsa-miR-886-3p |
| hsa-miR-328-4373049 |  | hsa-miR-10b |
| hsa-miR-27b-4373068 |  | hsa-miR-132 |
| hsa-miR-93*-002139 |  | hsa-miR-767-5p |
| hsa-miR-429-4373203 |  | hsa-miR-330-3p |
| hsa-miR-1290-002863 |  | hsa-miR-501-5p |
| hsa-miR-454-4395434 |  | hsa-miR-491-5p |
| hsa-miR-151-5P-002642 |  | hsa-miR-203 |
| hsa-miR-501-5p-4373226 |  | hsa-miR-339-3p |
| hsa-miR-532-3p-4395466 |  | hsa-miR-141 |
| hsa-miR-18a-4395533 |  | hsa-miR-362-5p |
| hsa-miR-301a-4373064 |  | hsa-miR-18a |
| hsa-miR-130b-4373144 |  | hsa-miR-320b |
| hsa-miR-103-4373158 |  | hsa-miR-429 |
| hsa-miR-660-4380925 |  | hsa-miR-378 |
| hsa-miR-766-001986 |  | hsa-miR-181a-2* |
| hsa-miR-330-3p-4373047 |  | hsa-miR-34a |
| hsa-miR-324-5p-4373052 |  | hsa-miR-145 |
| hsa-miR-378-002243 |  | hsa-miR-28-5p |
| hsa-miR-494-4395476 |  | hsa-miR-193b* |
| hsa-miR-423-5p-4395451 |  | hsa-miR-425* |
| hsa-miR-18a*-002423 |  | hsa-miR-146b-5p |
| hsa-miR-146b-5p-4373178 |  | hsa-miR-1201 |
| hsa-miR-148a-4373130 |  | hsa-miR-941 |
| hsa-miR-26b-4395167 |  | rno-miR-7* |
| hsa-miR-34a-4395168 |  | hsa-miR-10b* |
| hsa-miR-139-5p-4395400 |  | hsa-miR-26b |
| hsa-miR-34b-002102 |  | hsa-miR-34b |
| hsa-miR-128-4395327 |  | hsa-miR-660 |
| hsa-miR-124 |  | hsa-miR-25* |
| hsa-miR-224-4395210 |  | hsa-miR-195 |
| hsa-miR-193b*-002366 |  | hsa-miR-183 |
| hsa-miR-512-3p-4381034 |  | hsa-miR-92a-1* |
| hsa-miR-125a-5p-4395309 |  | hsa-miR-140-5p |
| hsa-miR-181a-2*-002317 |  | hsa-miR-9 |
| hsa-miR-192-4373108 |  | hsa-miR-589 |
| hsa-miR-1201-002781 |  | hsa-miR-138 |
| hsa-miR-941-002183 |  | hsa-miR-27a* |
| hsa-miR-769-5p-001998 |  | hsa-miR-423-5p |
| hsa-miR-375-4373027 |  | hsa-miR-21 |
| hsa-miR-195-4373105 |  | hsa-miR-182 |
| hsa-miR-106b*-002380 |  | hsa-miR-128 |
| hsa-miR-339-3p-4395295 |  | hsa-miR-301a |
| hsa-miR-148b-4373129 |  | hsa-miR-769-5p |
| hsa-miR-140-5p-4373374 |  | hsa-miR-942 |
| hsa-miR-99b*-002196 |  | hsa-miR-192 |
| hsa-miR-451 |  | hsa-miR-19b-1* |
| hsa-miR-483-5p-4395449 |  | hsa-miR-19a |
| hsa-miR-22*-002301 |  | hsa-miR-1271 |
| hsa-miR-589-001543 |  | hsa-miR-29a |
| hsa-miR-590-5p-4395176 |  | hsa-miR-455-3p |
| hsa-miR-29a-4395223 |  | hsa-miR-185 |
| hsa-miR-335*-002185 |  | hsa-miR-335* |
| hsa-miR-425*-002302 |  | hsa-miR-374a |
| hsa-miR-361-5p-4373035 |  | hsa-miR-135b* |
| hsa-miR-196b-4395326 |  | hsa-miR-30d |
| hsa-miR-636-4395199 |  | hsa-miR-99a* |
| hsa-miR-10b*-002315 |  | hsa-miR-629 |
| hsa-miR-517a-4395513 |  | hsa-miR-139-5p |
| hsa-miR-500-4395539 |  | hsa-miR-590-5p |
| hsa-miR-182-4395445 |  | hsa-miR-194 |
| hsa-miR-335-4373045 |  | hsa-miR-148b |
| hsa-miR-374a-4373028 |  | hsa-miR-107 |
| hsa-miR-194-4373106 |  | hsa-miR-1227 |
| hsa-miR-31*-002113 |  | hsa-miR-636 |
| hsa-miR-362-5p-4378092 |  | hsa-miR-500 |
| hsa-miR-138-4395395 |  | hsa-miR-671-3p |
| hsa-miR-185-4395382 |  | hsa-miR-505* |
| hsa-miR-622-001553 |  | hsa-miR-200a* |
| hsa-miR-942-002187 |  | hsa-miR-191* |
| hsa-miR-27b*-002174 |  | hsa-miR-212 |
| hsa-miR-212-4373087 |  | hsa-miR-34a* |
| hsa-miR-135b*-002159 |  | hsa-miR-361-5p |
| hsa-miR-455-3p-4395355 |  | hsa-miR-1254 |
| hsa-miR-183-4395380 |  | hsa-miR-652 |
| hsa-miR-27a*-002445 |  | hsa-miR-744* |
| hsa-miR-1303-002792 |  | hsa-miR-518e |
| hsa-miR-1271-002779 |  | hsa-miR-148b* |
| hsa-miR-301b-4395503 |  | hsa-miR-628-3p |
| hsa-miR-340*-002259 |  | hsa-miR-375 |
| hsa-miR-577-002675 |  | hsa-miR-577 |
| hsa-miR-126*-000451 |  | hsa-miR-125a-3p |
| hsa-miR-152-4395170 |  | hsa-miR-148a |
| hsa-miR-629-001562 |  | hsa-miR-642 |
| hsa-miR-517c-4373264 |  | hsa-miR-1290 |
| hsa-miR-671-3p-4395433 |  | hsa-miR-494 |
| hsa-miR-99a*-002141 |  | hsa-miR-452 |
| hsa-miR-505*-002087 |  | hsa-miR-335 |
| hsa-miR-891a-4395302 |  | hsa-miR-31* |
| hsa-miR-19b-1*-002425 |  | hsa-miR-10a* |
| hsa-miR-122 |  | hsa-miR-22* |
| dme-miR-7-000268 |  | hsa-miR-454* |
| hsa-miR-744*-002325 |  | hsa-miR-224 |
| hsa-miR-34a*-002316 |  | hsa-miR-26b* |
| hsa-miR-222* |  | hsa-miR-512-3p |
| hsa-miR-590-3P-002677 |  | hsa-miR-126* |
| hsa-miR-454*-001996 |  | hsa-miR-106b* |
| hsa-miR-519a-4395526 |  | hsa-miR-622 |
| hsa-miR-323-3p-4395338 |  | hsa-miR-338-5p |
| hsa-miR-1244-002791 |  | hsa-miR-483-5p |
| hsa-miR-1248 |  | hsa-miR-187 |
| hsa-miR-191*-002678 |  | hsa-miR-517c |
| hsa-miR-1300-002902 |  | hsa-miR-520D-3p |
| hsa-miR-601-001558 |  | hsa-miR-550 |
| hsa-miR-642-4380995 |  | hsa-miR-150 |
| hsa-miR-616-001589 |  | hsa-miR-152 |
| hsa-miR-92a-1*-002137 |  | hsa-miR-517a |
| hsa-miR-107-4373154 |  | hsa-miR-301b |
| hsa-miR-148b*-002160 |  | hsa-miR-202 |
| hsa-miR-452-4395440 |  | hsa-miR-340* |
| hsa-miR-652-4395463 |  | hsa-miR-891a |
| hsa-miR-520h |  | hsa-miR-140-3p |
| hsa-miR-338-5P-002658 |  | hsa-miR-20a* |
| hsa-miR-200a*-001011 |  | hsa-miR-1300 |
| hsa-miR-522-4395524 |  | hsa-miR-184 |
| hsa-miR-184-4373113 |  | hsa-miR-875-5p |
| hsa-miR-589-4395520 |  | hsa-miR-590-3p |
| hsa-miR-628-3p-002434 |  | hsa-miR-181c |
| hsa-miR-135a-4373140 |  | hsa-miR-101 |
| hsa-miR-520D-3P-002743 |  | hsa-miR-601 |
| hsa-miR-489-4395469 |  | hsa-miR-489 |
| hsa-miR-1227-002769 |  | hsa-miR-1303 |
| hsa-miR-193a-5p-4395392 |  | hsa-miR-502-3p |
| hsa-miR-518e-4395506 |  | hsa-miR-519a |
| hsa-miR-550-001544 |  | hsa-miR-1291 |
| hsa-miR-125a-3p-4395310 |  | hsa-miR-135a |
| hsa-miR-10a*-002288 |  | hsa-miR-323-3p |
| hsa-miR-1254-002818 |  | hsa-miR-522 |
| hsa-miR-181c-4373115 |  | hsa-miR-190b |
| hsa-miR-129* |  | hsa-miR-616 |
| hsa-miR-20a*-002437 |  | hsa-miR-193a-5p |
| hsa-miR-432 |  | hsa-miR-96 |
| hsa-miR-409-3p |  | hsa-miR-223 |
| hsa-miR-502-3p-4395194 |  | hsa-miR-1244 |
| hsa-miR-372-4373029 |  | hsa-miR-372 |
| hsa-let-7f-2* |  | hsa-miR-29c |
| hsa-miR-96-4373372 |  | hsa-miR-589 |
| hsa-miR-645-001597 |  | hsa-miR-628-5p |
| hsa-miR-26b*-002444 |  | hsa-miR-645 |
| hsa-miR-875-5p-002203 |  |  |
| hsa-miR-142-3p |  |  |
| hsa-miR-29c-4395171 |  |  |
| hsa-miR-140-3p-4395345 |  |  |
| hsa-let-7e* |  |  |
| hsa-miR-376c |  |  |
| hsa-miR-190b-002263 |  |  |
| hsa-miR-101-4395364 |  |  |
| hsa-miR-628-5p-4395544 |  |  |

Shading represents unique miRNAs
